# Supplementary material for: Hot spot prediction in protein-protein interactions by an ensemble system
Source: BMC Syst Biol. 2018 Dec 31;12(Suppl 9):132. doi: 10.1186/s12918-018-0665-8 (PMC6311905; doi:10.1186/s12918-018-0665-8)
Supplement: Supplementary file 2 — All relevant MATLAB code. A simple Matlab implement of our predictor. (ZIP 19,742 kb) [file 12918_2018_665_MOESM2_ESM.zip › Additional file 2/Read ASA/ASA website/dbMPIKT/4L72A.html]

 NetSurfP - Protein Surface Accessibility and Secondary Structure Predictions

  

|  |  |  |
| --- | --- | --- |
|  |  | NetSurfP - Protein Surface Accessibility and Secondary Structure Predictions  Technical University of Denmark |

  


---

```
# For publication of results, please cite:
# A generic method for assignment of reliability scores applied to solvent accessibility predictions.
# Bent Petersen, Thomas Nordahl Petersen, Pernille Andersen, Morten Nielsen and Claus Lundegaard
# BMC Structural Biology 2009, 9:51 doi:10.1186/1472-6807-9-51
#
# Column 1: Class assignment - B for buried or E for Exposed - Threshold: 25% exposure, but not based on RSA
# Column 2: Amino acid
# Column 3: Sequence name
# Column 4: Amino acid number
# Column 5: Relative Surface Accessibility - RSA
# Column 6: Absolute Surface Accessibility
# Column 7: Z-fit score for RSA prediction
# Column 8: Probability for Alpha-Helix
# Column 9: Probability for Beta-strand
# Column 10: Probability for Coil
E S  4L72A                  1    0.913 107.039   0.426   0.003   0.003   0.994
E R  4L72A                  2    0.780 178.551   0.489   0.005   0.015   0.979
E K  4L72A                  3    0.536 110.193   0.480   0.018   0.088   0.893
E T  4L72A                  4    0.571  79.128   1.681   0.021   0.279   0.699
B Y  4L72A                  5    0.075  16.028   0.591   0.022   0.359   0.619
E T  4L72A                  6    0.340  47.158   1.967   0.074   0.484   0.442
B L  4L72A                  7    0.104  19.061   0.308   0.159   0.516   0.325
E T  4L72A                  8    0.442  61.361   0.458   0.079   0.592   0.329
E D  4L72A                  9    0.383  55.133   0.381   0.079   0.592   0.329
B Y  4L72A                 10    0.103  22.054  -0.466   0.069   0.386   0.545
B L  4L72A                 11    0.187  34.166   0.601   0.066   0.296   0.638
E K  4L72A                 12    0.502 103.364   0.172   0.018   0.088   0.893
E N  4L72A                 13    0.483  70.682  -1.657   0.018   0.047   0.935
E T  4L72A                 14    0.589  81.722   0.267   0.020   0.205   0.775
B Y  4L72A                 15    0.156  33.359  -1.172   0.021   0.279   0.699
E R  4L72A                 16    0.322  73.853   0.284   0.020   0.205   0.775
B L  4L72A                 17    0.244  44.731  -1.712   0.020   0.205   0.775
E K  4L72A                 18    0.613 126.176  -0.268   0.020   0.205   0.775
E L  4L72A                 19    0.417  76.389  -1.594   0.004   0.197   0.799
B Y  4L72A                 20    0.278  59.451  -0.344   0.005   0.262   0.733
E S  4L72A                 21    0.423  49.587  -0.875   0.005   0.262   0.733
B L  4L72A                 22    0.073  13.293  -1.023   0.005   0.336   0.660
E R  4L72A                 23    0.402  92.081   0.684   0.023   0.655   0.322
B W  4L72A                 24    0.131  31.626  -1.982   0.023   0.655   0.322
B I  4L72A                 25    0.146  26.991  -0.548   0.005   0.336   0.660
E S  4L72A                 26    0.521  61.073  -0.365   0.004   0.085   0.910
E D  4L72A                 27    0.594  85.653  -1.096   0.018   0.019   0.964
E H  4L72A                 28    0.427  77.744  -0.976   0.004   0.138   0.858
E E  4L72A                 29    0.465  81.236   0.299   0.004   0.616   0.381
B Y  4L72A                 30    0.128  27.396   0.429   0.001   0.900   0.099
B L  4L72A                 31    0.139  25.378   0.510   0.001   0.900   0.099
B Y  4L72A                 32    0.177  37.782   0.198   0.001   0.900   0.099
E K  4L72A                 33    0.356  73.126   0.883   0.002   0.816   0.182
E Q  4L72A                 34    0.530  94.712   0.387   0.005   0.262   0.733
E E  4L72A                 35    0.685 119.722  -0.993   0.005   0.015   0.979
E N  4L72A                 36    0.587  85.893  -1.215   0.005   0.045   0.951
B N  4L72A                 37    0.219  32.062  -0.083   0.004   0.514   0.481
B I  4L72A                 38    0.080  14.837   0.138   0.001   0.900   0.099
B L  4L72A                 39    0.077  14.154   0.696   0.001   0.959   0.040
B V  4L72A                 40    0.090  13.787   0.483   0.001   0.959   0.040
B F  4L72A                 41    0.065  13.025   0.858   0.001   0.959   0.040
B N  4L72A                 42    0.156  22.794   0.472   0.001   0.900   0.099
B A  4L72A                 43    0.084   9.312   0.087   0.004   0.420   0.576
E E  4L72A                 44    0.567  99.020   0.730   0.004   0.197   0.799
E Y  4L72A                 45    0.563 120.249   0.337   0.018   0.047   0.935
E G  4L72A                 46    0.486  38.264  -0.766   0.018   0.019   0.964
E N  4L72A                 47    0.588  86.112   0.860   0.021   0.279   0.699
E S  4L72A                 48    0.462  54.088   1.068   0.023   0.655   0.322
E S  4L72A                 49    0.428  50.197   1.374   0.021   0.756   0.223
E V  4L72A                 50    0.322  49.445   1.308   0.021   0.756   0.223
B F  4L72A                 51    0.126  25.228   0.089   0.079   0.592   0.329
B L  4L72A                 52    0.236  43.193   0.240   0.159   0.516   0.325
E E  4L72A                 53    0.323  56.463   0.025   0.231   0.330   0.439
E N  4L72A                 54    0.369  53.963  -0.409   0.605   0.105   0.290
E S  4L72A                 55    0.516  60.475  -0.161   0.660   0.049   0.291
E T  4L72A                 56    0.285  39.516   0.694   0.701   0.107   0.192
B F  4L72A                 57    0.136  27.295   0.004   0.561   0.047   0.393
E D  4L72A                 58    0.397  57.222  -0.103   0.561   0.047   0.393
E E  4L72A                 59    0.573 100.103  -0.175   0.455   0.046   0.498
E F  4L72A                 60    0.360  72.332  -0.174   0.354   0.048   0.598
E G  4L72A                 61    0.378  29.725  -1.105   0.113   0.043   0.844
E H  4L72A                 62    0.478  86.894  -0.054   0.113   0.043   0.844
E S  4L72A                 63    0.352  41.208   0.398   0.064   0.216   0.721
B I  4L72A                 64    0.044   8.085  -1.160   0.021   0.451   0.528
E N  4L72A                 65    0.331  48.488  -0.696   0.021   0.451   0.528
E D  4L72A                 66    0.393  56.689  -0.175   0.021   0.451   0.528
B Y  4L72A                 67    0.150  31.970   0.583   0.023   0.655   0.322
E S  4L72A                 68    0.394  46.212   0.281   0.018   0.846   0.136
B I  4L72A                 69    0.168  31.117  -0.262   0.002   0.816   0.182
B S  4L72A                 70    0.082   9.646  -0.150   0.005   0.336   0.660
E P  4L72A                 71    0.691  98.110   0.271   0.005   0.045   0.951
E D  4L72A                 72    0.586  84.443   0.728   0.005   0.015   0.979
E G  4L72A                 73    0.350  27.514  -0.385   0.018   0.019   0.964
E Q  4L72A                 74    0.494  88.282   1.062   0.004   0.085   0.910
B F  4L72A                 75    0.233  46.703   1.682   0.002   0.816   0.182
B I  4L72A                 76    0.022   3.996   1.303   0.001   0.959   0.040
B L  4L72A                 77    0.044   8.020   0.577   0.001   0.959   0.040
B L  4L72A                 78    0.042   7.672   0.770   0.000   0.983   0.017
B E  4L72A                 79    0.050   8.770   0.385   0.001   0.959   0.040
B Y  4L72A                 80    0.097  20.772   0.686   0.001   0.959   0.040
E N  4L72A                 81    0.308  45.091  -0.078   0.002   0.816   0.182
E Y  4L72A                 82    0.286  61.118  -0.032   0.022   0.552   0.426
E V  4L72A                 83    0.431  66.306  -0.409   0.022   0.552   0.426
E K  4L72A                 84    0.551 113.238  -0.968   0.022   0.359   0.619
E Q  4L72A                 85    0.403  72.029  -1.333   0.021   0.279   0.699
E W  4L72A                 86    0.387  93.122  -1.189   0.021   0.279   0.699
E R  4L72A                 87    0.493 112.805  -0.675   0.021   0.279   0.699
E H  4L72A                 88    0.325  59.208  -1.563   0.021   0.279   0.699
E S  4L72A                 89    0.431  50.490  -1.292   0.005   0.262   0.733
B Y  4L72A                 90    0.275  58.874  -1.297   0.004   0.420   0.576
E T  4L72A                 91    0.308  42.761  -0.003   0.004   0.616   0.381
B A  4L72A                 92    0.050   5.477  -0.822   0.004   0.616   0.381
B S  4L72A                 93    0.174  20.428  -0.027   0.002   0.816   0.182
B Y  4L72A                 94    0.045   9.552   0.525   0.001   0.959   0.040
B D  4L72A                 95    0.073  10.534   0.739   0.001   0.959   0.040
B I  4L72A                 96    0.036   6.605  -0.406   0.001   0.959   0.040
B Y  4L72A                 97    0.104  22.310   1.040   0.001   0.959   0.040
B D  4L72A                 98    0.231  33.215   0.053   0.002   0.816   0.182
B L  4L72A                 99    0.163  29.827   0.059   0.018   0.088   0.893
E N  4L72A                100    0.710 103.988   0.445   0.005   0.015   0.979
E K  4L72A                101    0.653 134.343  -0.740   0.016   0.005   0.979
E R  4L72A                102    0.542 124.118  -0.941   0.018   0.019   0.964
E Q  4L72A                103    0.582 103.874   0.601   0.022   0.359   0.619
E L  4L72A                104    0.260  47.514   0.092   0.021   0.756   0.223
E I  4L72A                105    0.367  67.803   0.542   0.021   0.756   0.223
E T  4L72A                106    0.441  61.208   0.416   0.023   0.655   0.322
B E  4L72A                107    0.141  24.563  -0.497   0.021   0.451   0.528
E E  4L72A                108    0.420  73.287  -1.119   0.020   0.205   0.775
E R  4L72A                109    0.564 129.042  -1.313   0.019   0.141   0.840
E I  4L72A                110    0.406  75.110  -0.954   0.018   0.047   0.935
E P  4L72A                111    0.645  91.596  -1.534   0.018   0.019   0.964
E N  4L72A                112    0.553  80.886  -1.488   0.018   0.047   0.935
E N  4L72A                113    0.509  74.532  -0.469   0.056   0.142   0.802
B T  4L72A                114    0.215  29.834  -1.268   0.022   0.359   0.619
E Q  4L72A                115    0.326  58.241  -0.866   0.021   0.451   0.528
B W  4L72A                116    0.268  64.334  -0.379   0.021   0.451   0.528
B V  4L72A                117    0.194  29.741  -0.074   0.022   0.552   0.426
E T  4L72A                118    0.300  41.652  -0.568   0.023   0.655   0.322
B W  4L72A                119    0.069  16.667  -1.189   0.022   0.552   0.426
B S  4L72A                120    0.057   6.680  -0.699   0.004   0.138   0.858
E P  4L72A                121    0.455  64.593  -0.653   0.004   0.085   0.910
E V  4L72A                122    0.466  71.686   0.047   0.018   0.019   0.964
E G  4L72A                123    0.295  23.256  -1.150   0.018   0.019   0.964
E H  4L72A                124    0.443  80.618   0.464   0.004   0.138   0.858
B K  4L72A                125    0.139  28.613   1.306   0.002   0.816   0.182
B L  4L72A                126    0.021   3.900   1.379   0.001   0.900   0.099
B A  4L72A                127    0.026   2.898   1.012   0.001   0.959   0.040
B Y  4L72A                128    0.043   9.082   0.205   0.001   0.959   0.040
B V  4L72A                129    0.026   3.919   0.569   0.001   0.959   0.040
B W  4L72A                130    0.130  31.265   0.690   0.001   0.900   0.099
E N  4L72A                131    0.387  56.628   0.227   0.004   0.197   0.799
E N  4L72A                132    0.430  62.908  -0.524   0.005   0.015   0.979
E D  4L72A                133    0.338  48.677  -0.842   0.005   0.262   0.733
B I  4L72A                134    0.101  18.648  -0.091   0.003   0.718   0.279
B Y  4L72A                135    0.139  29.619   0.270   0.018   0.846   0.136
B V  4L72A                136    0.129  19.873  -0.179   0.018   0.846   0.136
B K  4L72A                137    0.216  44.452   0.545   0.021   0.756   0.223
E I  4L72A                138    0.512  94.776  -0.687   0.023   0.655   0.322
B E  4L72A                139    0.224  39.220  -0.239   0.019   0.141   0.840
E P  4L72A                140    0.704  99.855  -1.205   0.018   0.019   0.964
E N  4L72A                141    0.707 103.519  -0.502   0.018   0.019   0.964
E L  4L72A                142    0.514  94.113  -1.114   0.018   0.047   0.935
E P  4L72A                143    0.555  78.769   0.262   0.005   0.262   0.733
E S  4L72A                144    0.399  46.810   0.087   0.003   0.718   0.279
E Y  4L72A                145    0.274  58.490   1.332   0.001   0.900   0.099
E R  4L72A                146    0.367  83.974   1.002   0.001   0.900   0.099
B I  4L72A                147    0.107  19.739   0.618   0.001   0.900   0.099
B T  4L72A                148    0.135  18.724   0.059   0.003   0.718   0.279
E W  4L72A                149    0.394  94.781  -0.219   0.004   0.420   0.576
E T  4L72A                150    0.564  78.268  -0.511   0.018   0.047   0.935
E G  4L72A                151    0.582  45.827  -2.384   0.058   0.017   0.925
E K  4L72A                152    0.527 108.507  -0.982   0.053   0.043   0.903
E E  4L72A                153    0.564  98.566  -0.545   0.052   0.084   0.864
E D  4L72A                154    0.450  64.773  -0.798   0.064   0.216   0.721
E I  4L72A                155    0.334  61.716   0.027   0.079   0.592   0.329
E I  4L72A                156    0.273  50.524  -0.503   0.021   0.756   0.223
B Y  4L72A                157    0.208  44.385  -0.513   0.023   0.655   0.322
E N  4L72A                158    0.347  50.801  -1.455   0.069   0.386   0.545
E G  4L72A                159    0.376  29.591  -1.316   0.064   0.216   0.721
E I  4L72A                160    0.286  52.984  -0.525   0.056   0.142   0.802
E T  4L72A                161    0.407  56.437  -1.439   0.113   0.087   0.800
E D  4L72A                162    0.567  81.705  -1.730   0.113   0.087   0.800
B W  4L72A                163    0.236  56.806  -0.656   0.199   0.152   0.649
B V  4L72A                164    0.226  34.813  -1.250   0.216   0.235   0.548
E Y  4L72A                165    0.295  63.106  -0.264   0.216   0.235   0.548
E E  4L72A                166    0.522  91.246  -0.756   0.199   0.152   0.649
E E  4L72A                167    0.439  76.693  -1.273   0.199   0.152   0.649
E E  4L72A                168    0.493  86.197  -0.706   0.125   0.227   0.648
B V  4L72A                169    0.227  34.921  -0.403   0.069   0.386   0.545
B F  4L72A                170    0.252  50.556  -0.816   0.074   0.484   0.442
E S  4L72A                171    0.362  42.391  -1.020   0.022   0.552   0.426
E A  4L72A                172    0.309  34.074  -0.720   0.021   0.451   0.528
B Y  4L72A                173    0.155  33.209  -1.169   0.021   0.451   0.528
E S  4L72A                174    0.283  33.156  -0.498   0.021   0.451   0.528
B A  4L72A                175    0.080   8.761  -1.390   0.021   0.756   0.223
B L  4L72A                176    0.078  14.355  -0.412   0.018   0.846   0.136
B W  4L72A                177    0.147  35.281  -0.851   0.018   0.846   0.136
B W  4L72A                178    0.104  24.940  -0.548   0.002   0.816   0.182
B S  4L72A                179    0.053   6.223  -0.611   0.005   0.336   0.660
E P  4L72A                180    0.480  68.155  -0.760   0.004   0.085   0.910
E N  4L72A                181    0.495  72.424  -0.541   0.005   0.015   0.979
E G  4L72A                182    0.313  24.641  -1.234   0.005   0.015   0.979
E T  4L72A                183    0.389  53.968   0.421   0.004   0.138   0.858
B F  4L72A                184    0.138  27.677   1.361   0.003   0.718   0.279
B L  4L72A                185    0.021   3.918   1.303   0.001   0.900   0.099
B A  4L72A                186    0.024   2.678   0.972   0.001   0.959   0.040
B Y  4L72A                187    0.029   6.133   0.302   0.001   0.959   0.040
B A  4L72A                188    0.035   3.835  -0.439   0.001   0.959   0.040
B Q  4L72A                189    0.114  20.289  -0.554   0.001   0.959   0.040
B F  4L72A                190    0.159  31.831  -0.380   0.002   0.816   0.182
E N  4L72A                191    0.401  58.648  -0.422   0.004   0.420   0.576
E D  4L72A                192    0.586  84.385  -1.276   0.018   0.047   0.935
E T  4L72A                193    0.572  79.323  -1.860   0.018   0.019   0.964
E E  4L72A                194    0.671 117.136  -1.328   0.018   0.047   0.935
E V  4L72A                195    0.351  53.903  -0.282   0.018   0.088   0.893
E P  4L72A                196    0.480  68.155  -0.412   0.021   0.279   0.699
E L  4L72A                197    0.443  81.168  -0.157   0.023   0.655   0.322
B I  4L72A                198    0.130  24.050   0.192   0.021   0.756   0.223
E E  4L72A                199    0.425  74.265  -0.334   0.021   0.756   0.223
B Y  4L72A                200    0.141  30.217   0.520   0.023   0.655   0.322
E S  4L72A                201    0.309  36.250  -0.443   0.022   0.552   0.426
E F  4L72A                202    0.331  66.492  -0.563   0.069   0.386   0.545
E Y  4L72A                203    0.330  70.542  -1.072   0.066   0.296   0.638
E S  4L72A                204    0.398  46.646  -0.941   0.113   0.087   0.800
E D  4L72A                205    0.592  85.293  -1.148   0.113   0.043   0.844
E E  4L72A                206    0.657 114.848  -0.843   0.115   0.016   0.868
E S  4L72A                207    0.411  48.146  -1.447   0.113   0.043   0.844
E L  4L72A                208    0.494  90.506  -0.558   0.113   0.087   0.800
E Q  4L72A                209    0.472  84.263  -0.152   0.118   0.150   0.732
B Y  4L72A                210    0.174  37.248  -0.171   0.118   0.150   0.732
E P  4L72A                211    0.470  66.707  -0.922   0.056   0.142   0.802
E K  4L72A                212    0.535 110.132  -0.005   0.066   0.296   0.638
E T  4L72A                213    0.304  42.220  -0.845   0.074   0.484   0.442
E V  4L72A                214    0.326  50.122   0.170   0.079   0.592   0.329
E R  4L72A                215    0.445 101.951  -0.406   0.087   0.683   0.230
B V  4L72A                216    0.146  22.502   0.394   0.087   0.683   0.230
E P  4L72A                217    0.430  61.060  -0.454   0.079   0.592   0.329
B Y  4L72A                218    0.158  33.743  -0.743   0.069   0.386   0.545
E P  4L72A                219    0.366  51.879  -1.227   0.125   0.227   0.648
E K  4L72A                220    0.400  82.342  -0.805   0.125   0.227   0.648
E A  4L72A                221    0.379  41.777  -0.974   0.125   0.227   0.648
E G  4L72A                222    0.413  32.511  -1.258   0.125   0.227   0.648
E A  4L72A                223    0.359  39.540  -0.887   0.056   0.142   0.802
E V  4L72A                224    0.582  89.484  -1.253   0.053   0.043   0.903
E N  4L72A                225    0.308  45.150  -1.093   0.018   0.019   0.964
E P  4L72A                226    0.466  66.125  -1.139   0.005   0.045   0.951
E T  4L72A                227    0.411  57.047   0.504   0.004   0.420   0.576
B V  4L72A                228    0.071  10.928  -0.610   0.003   0.718   0.279
B K  4L72A                229    0.146  30.114   0.729   0.002   0.816   0.182
B F  4L72A                230    0.033   6.583  -0.261   0.001   0.959   0.040
B F  4L72A                231    0.041   8.229   0.662   0.001   0.959   0.040
B V  4L72A                232    0.039   6.071  -0.616   0.001   0.959   0.040
B V  4L72A                233    0.042   6.517   0.226   0.001   0.959   0.040
B N  4L72A                234    0.179  26.206  -0.353   0.002   0.816   0.182
B T  4L72A                235    0.104  14.397  -0.517   0.020   0.205   0.775
E D  4L72A                236    0.655  94.414  -0.299   0.018   0.047   0.935
E S  4L72A                237    0.619  72.547  -0.502   0.018   0.019   0.964
E L  4L72A                238    0.427  78.110  -1.281   0.018   0.047   0.935
E S  4L72A                239    0.541  63.382  -0.748   0.022   0.359   0.619
E S  4L72A                240    0.439  51.416  -0.218   0.021   0.756   0.223
E V  4L72A                241    0.312  47.924  -0.406   0.021   0.756   0.223
E T  4L72A                242    0.354  49.086  -0.430   0.021   0.756   0.223
B N  4L72A                243    0.279  40.904  -0.298   0.022   0.552   0.426
E A  4L72A                244    0.325  35.771  -1.210   0.022   0.359   0.619
E T  4L72A                245    0.382  52.928  -0.426   0.066   0.296   0.638
E S  4L72A                246    0.459  53.736  -1.051   0.064   0.216   0.721
E I  4L72A                247    0.330  61.069  -1.525   0.125   0.227   0.648
E Q  4L72A                248    0.485  86.567  -1.347   0.125   0.227   0.648
B I  4L72A                249    0.216  39.960  -1.581   0.125   0.227   0.648
E T  4L72A                250    0.455  63.164  -1.377   0.199   0.152   0.649
E A  4L72A                251    0.403  44.466  -0.956   0.113   0.087   0.800
B P  4L72A                252    0.271  38.455  -1.519   0.113   0.087   0.800
E A  4L72A                253    0.439  48.400  -2.048   0.113   0.087   0.800
E S  4L72A                254    0.475  55.682  -1.309   0.113   0.087   0.800
E M  4L72A                255    0.315  63.052  -1.858   0.113   0.087   0.800
E L  4L72A                256    0.435  79.722  -1.214   0.052   0.084   0.864
E I  4L72A                257    0.507  93.795  -1.493   0.053   0.043   0.903
E G  4L72A                258    0.632  49.715  -0.980   0.018   0.047   0.935
E D  4L72A                259    0.417  60.133  -1.101   0.019   0.141   0.840
B H  4L72A                260    0.223  40.582  -0.755   0.004   0.420   0.576
B Y  4L72A                261    0.162  34.726  -0.588   0.004   0.616   0.381
B L  4L72A                262    0.054   9.961   0.193   0.003   0.718   0.279
B C  4L72A                263    0.067   9.365  -1.113   0.002   0.816   0.182
B D  4L72A                264    0.138  19.871  -0.767   0.002   0.816   0.182
B V  4L72A                265    0.038   5.856  -1.260   0.002   0.816   0.182
B T  4L72A                266    0.222  30.764  -0.769   0.001   0.900   0.099
B W  4L72A                267    0.125  30.062  -0.241   0.002   0.816   0.182
E A  4L72A                268    0.232  25.522  -0.537   0.004   0.420   0.576
E T  4L72A                269    0.548  76.021  -0.883   0.004   0.085   0.910
E Q  4L72A                270    0.691 123.502  -1.133   0.003   0.003   0.994
E E  4L72A                271    0.474  82.808  -1.394   0.005   0.015   0.979
E R  4L72A                272    0.378  86.608   0.026   0.005   0.336   0.660
B I  4L72A                273    0.112  20.794   0.328   0.002   0.816   0.182
B S  4L72A                274    0.040   4.688   0.489   0.001   0.959   0.040
B L  4L72A                275    0.022   3.937   1.217   0.001   0.959   0.040
B Q  4L72A                276    0.043   7.733   0.415   0.000   0.983   0.017
B W  4L72A                277    0.033   8.057  -0.685   0.001   0.959   0.040
B L  4L72A                278    0.052   9.576  -0.488   0.001   0.900   0.099
B R  4L72A                279    0.244  55.968  -0.315   0.003   0.718   0.279
E R  4L72A                280    0.281  64.441   0.093   0.005   0.262   0.733
E I  4L72A                281    0.526  97.292  -1.462   0.004   0.085   0.910
E Q  4L72A                282    0.549  98.087  -0.742   0.005   0.045   0.951
E N  4L72A                283    0.589  86.171  -1.212   0.005   0.045   0.951
E Y  4L72A                284    0.324  69.153  -0.310   0.005   0.336   0.660
E S  4L72A                285    0.397  46.528  -0.170   0.004   0.616   0.381
B V  4L72A                286    0.185  28.450   0.621   0.002   0.816   0.182
B M  4L72A                287    0.050   9.985   0.203   0.001   0.900   0.099
B D  4L72A                288    0.097  13.963   0.520   0.001   0.959   0.040
B I  4L72A                289    0.029   5.309   0.400   0.001   0.959   0.040
B C  4L72A                290    0.052   7.301   0.908   0.001   0.959   0.040
B D  4L72A                291    0.139  19.972  -0.072   0.001   0.900   0.099
B Y  4L72A                292    0.106  22.609   0.293   0.004   0.616   0.381
E D  4L72A                293    0.373  53.807   0.188   0.005   0.262   0.733
E E  4L72A                294    0.342  59.800  -0.885   0.018   0.019   0.964
E S  4L72A                295    0.714  83.728  -1.036   0.018   0.019   0.964
E S  4L72A                296    0.500  58.565  -1.147   0.018   0.019   0.964
E G  4L72A                297    0.423  33.290  -0.912   0.018   0.088   0.893
E R  4L72A                298    0.519 118.782  -0.044   0.021   0.451   0.528
E W  4L72A                299    0.257  61.784   0.232   0.021   0.756   0.223
E N  4L72A                300    0.359  52.602   0.189   0.021   0.756   0.223
E C  4L72A                301    0.286  40.197   0.133   0.023   0.655   0.322
B L  4L72A                302    0.163  29.845  -0.075   0.022   0.552   0.426
B V  4L72A                303    0.181  27.804  -0.404   0.074   0.484   0.442
E A  4L72A                304    0.405  44.620  -0.479   0.066   0.296   0.638
E R  4L72A                305    0.528 120.866  -0.629   0.118   0.150   0.732
E Q  4L72A                306    0.586 104.624  -0.392   0.191   0.086   0.723
E H  4L72A                307    0.383  69.595  -0.708   0.191   0.086   0.723
E I  4L72A                308    0.310  57.332  -0.669   0.191   0.086   0.723
E E  4L72A                309    0.585 102.287  -0.816   0.199   0.152   0.649
E M  4L72A                310    0.378  75.718  -0.606   0.199   0.152   0.649
E S  4L72A                311    0.340  39.825  -1.558   0.199   0.152   0.649
E T  4L72A                312    0.398  55.133  -0.897   0.199   0.152   0.649
E T  4L72A                313    0.540  74.884  -0.954   0.191   0.086   0.723
E G  4L72A                314    0.364  28.686  -0.627   0.199   0.152   0.649
B W  4L72A                315    0.231  55.676  -1.266   0.199   0.152   0.649
B V  4L72A                316    0.181  27.820  -1.267   0.199   0.152   0.649
E G  4L72A                317    0.433  34.116  -1.690   0.113   0.087   0.800
E R  4L72A                318    0.336  76.990  -1.277   0.113   0.087   0.800
B F  4L72A                319    0.261  52.363  -1.751   0.113   0.087   0.800
E R  4L72A                320    0.390  89.196  -0.696   0.052   0.084   0.864
B P  4L72A                321    0.285  40.456  -2.002   0.056   0.142   0.802
E S  4L72A                322    0.314  36.754  -1.602   0.064   0.216   0.721
B E  4L72A                323    0.261  45.509  -0.518   0.021   0.279   0.699
B P  4L72A                324    0.141  20.022  -0.642   0.021   0.451   0.528
B H  4L72A                325    0.251  45.693  -0.446   0.023   0.655   0.322
B F  4L72A                326    0.157  31.510  -1.330   0.021   0.756   0.223
B T  4L72A                327    0.071   9.917  -1.590   0.021   0.451   0.528
E L  4L72A                328    0.483  88.474  -0.652   0.004   0.197   0.799
E D  4L72A                329    0.500  72.064  -0.864   0.005   0.045   0.951
E G  4L72A                330    0.447  35.179  -1.682   0.005   0.015   0.979
E N  4L72A                331    0.432  63.216  -0.027   0.004   0.085   0.910
B S  4L72A                332    0.178  20.815   0.481   0.003   0.718   0.279
B F  4L72A                333    0.031   6.322   0.562   0.001   0.900   0.099
B Y  4L72A                334    0.033   7.009   0.571   0.001   0.959   0.040
B K  4L72A                335    0.038   7.858  -0.139   0.001   0.959   0.040
B I  4L72A                336    0.043   7.973   0.019   0.001   0.959   0.040
B I  4L72A                337    0.065  11.951  -0.666   0.002   0.816   0.182
B S  4L72A                338    0.188  22.080  -0.361   0.004   0.616   0.381
E N  4L72A                339    0.449  65.660   0.376   0.004   0.197   0.799
E E  4L72A                340    0.636 111.022  -0.717   0.018   0.088   0.893
E E  4L72A                341    0.582 101.745  -1.837   0.005   0.015   0.979
E G  4L72A                342    0.547  43.080  -1.821   0.005   0.015   0.979
B Y  4L72A                343    0.212  45.390  -0.840   0.004   0.138   0.858
B R  4L72A                344    0.241  55.075  -0.292   0.004   0.420   0.576
B H  4L72A                345    0.118  21.391  -0.214   0.003   0.718   0.279
B I  4L72A                346    0.035   6.438  -0.411   0.001   0.900   0.099
B C  4L72A                347    0.057   8.031   0.774   0.001   0.959   0.040
B Y  4L72A                348    0.107  22.887  -0.118   0.001   0.959   0.040
B F  4L72A                349    0.070  14.069   0.563   0.001   0.959   0.040
B Q  4L72A                350    0.195  34.898  -0.164   0.023   0.655   0.322
B I  4L72A                351    0.241  44.567  -0.925   0.020   0.205   0.775
E D  4L72A                352    0.567  81.734  -1.460   0.018   0.047   0.935
E K  4L72A                353    0.589 121.240  -1.238   0.005   0.015   0.979
E K  4L72A                354    0.595 122.392  -1.370   0.018   0.019   0.964
E D  4L72A                355    0.593  85.495   0.119   0.020   0.205   0.775
E C  4L72A                356    0.291  40.786   0.471   0.022   0.552   0.426
E T  4L72A                357    0.452  62.665   0.637   0.023   0.655   0.322
E F  4L72A                358    0.371  74.520   0.807   0.023   0.655   0.322
B I  4L72A                359    0.078  14.338  -0.307   0.022   0.552   0.426
B T  4L72A                360    0.190  26.367  -0.207   0.021   0.279   0.699
E K  4L72A                361    0.552 113.485  -0.449   0.018   0.088   0.893
E G  4L72A                362    0.535  42.065  -1.330   0.018   0.019   0.964
E T  4L72A                363    0.563  78.046  -1.350   0.058   0.017   0.925
B W  4L72A                364    0.294  70.635  -0.971   0.052   0.084   0.864
E E  4L72A                365    0.410  71.662  -0.588   0.056   0.142   0.802
B V  4L72A                366    0.156  23.993  -1.210   0.066   0.296   0.638
B I  4L72A                367    0.264  48.895  -0.880   0.066   0.296   0.638
E G  4L72A                368    0.387  30.425  -0.793   0.066   0.296   0.638
B I  4L72A                369    0.111  20.443  -0.588   0.074   0.484   0.442
B E  4L72A                370    0.217  37.857  -0.770   0.022   0.552   0.426
E A  4L72A                371    0.299  32.906  -0.696   0.023   0.655   0.322
B L  4L72A                372    0.136  24.810  -0.593   0.021   0.756   0.223
E T  4L72A                373    0.241  33.427  -1.001   0.021   0.451   0.528
E S  4L72A                374    0.654  76.672  -1.602   0.005   0.045   0.951
E D  4L72A                375    0.670  96.547  -1.226   0.005   0.015   0.979
B Y  4L72A                376    0.235  50.305   0.012   0.005   0.262   0.733
B L  4L72A                377    0.137  25.121  -0.265   0.004   0.616   0.381
B Y  4L72A                378    0.092  19.618   0.793   0.001   0.900   0.099
B Y  4L72A                379    0.030   6.454   0.001   0.001   0.959   0.040
B I  4L72A                380    0.047   8.714  -0.060   0.001   0.959   0.040
B S  4L72A                381    0.033   3.856  -1.318   0.001   0.900   0.099
B N  4L72A                382    0.142  20.716   0.022   0.002   0.816   0.182
B E  4L72A                383    0.243  42.382  -0.708   0.004   0.616   0.381
E Y  4L72A                384    0.399  85.224  -0.803   0.022   0.359   0.619
E K  4L72A                385    0.518 106.491  -1.623   0.019   0.141   0.840
E G  4L72A                386    0.564  44.371  -1.655   0.019   0.141   0.840
E M  4L72A                387    0.367  73.497  -0.319   0.021   0.279   0.699
E P  4L72A                388    0.385  54.561  -0.658   0.022   0.359   0.619
E G  4L72A                389    0.325  25.609  -1.310   0.022   0.359   0.619
B G  4L72A                390    0.220  17.314  -0.686   0.004   0.514   0.481
B R  4L72A                391    0.221  50.632  -0.643   0.004   0.616   0.381
B N  4L72A                392    0.161  23.585  -0.111   0.002   0.816   0.182
B L  4L72A                393    0.105  19.244  -0.698   0.001   0.900   0.099
B Y  4L72A                394    0.088  18.763   0.079   0.011   0.918   0.071
B K  4L72A                395    0.216  44.328  -0.176   0.018   0.846   0.136
B I  4L72A                396    0.102  18.851   0.184   0.021   0.756   0.223
E Q  4L72A                397    0.329  58.759  -0.181   0.023   0.655   0.322
B L  4L72A                398    0.091  16.589  -0.998   0.020   0.205   0.775
E S  4L72A                399    0.621  72.758  -0.799   0.018   0.047   0.935
E D  4L72A                400    0.585  84.226  -1.216   0.018   0.019   0.964
E Y  4L72A                401    0.517 110.483  -1.393   0.018   0.019   0.964
E T  4L72A                402    0.569  78.948  -0.576   0.052   0.084   0.864
E K  4L72A                403    0.433  89.089   0.478   0.069   0.386   0.545
E V  4L72A                404    0.367  56.377   0.127   0.022   0.552   0.426
E T  4L72A                405    0.493  68.365   0.470   0.079   0.592   0.329
E C  4L72A                406    0.254  35.676  -0.272   0.079   0.592   0.329
E L  4L72A                407    0.283  51.872  -0.389   0.074   0.484   0.442
E S  4L72A                408    0.477  55.916  -0.716   0.066   0.296   0.638
B C  4L72A                409    0.232  32.587  -0.499   0.056   0.142   0.802
E E  4L72A                410    0.695 121.347  -1.147   0.052   0.084   0.864
E L  4L72A                411    0.325  59.562  -1.987   0.052   0.084   0.864
E N  4L72A                412    0.339  49.659  -1.363   0.052   0.084   0.864
E P  4L72A                413    0.408  57.952  -1.652   0.053   0.043   0.903
E E  4L72A                414    0.611 106.812  -2.068   0.018   0.047   0.935
E R  4L72A                415    0.463 106.027  -1.315   0.018   0.047   0.935
B C  4L72A                416    0.208  29.259  -0.951   0.004   0.197   0.799
B Q  4L72A                417    0.254  45.347  -0.985   0.004   0.514   0.481
B Y  4L72A                418    0.077  16.498  -0.709   0.002   0.816   0.182
B Y  4L72A                419    0.131  27.995  -0.597   0.002   0.816   0.182
B S  4L72A                420    0.100  11.767  -1.223   0.001   0.900   0.099
B V  4L72A                421    0.091  13.910  -0.560   0.002   0.816   0.182
B S  4L72A                422    0.189  22.139  -0.540   0.001   0.900   0.099
B F  4L72A                423    0.156  31.229  -0.333   0.003   0.718   0.279
B S  4L72A                424    0.154  18.061  -0.632   0.004   0.197   0.799
E K  4L72A                425    0.659 135.639  -0.235   0.005   0.015   0.979
E E  4L72A                426    0.662 115.634  -0.311   0.005   0.015   0.979
B A  4L72A                427    0.268  29.534  -0.552   0.018   0.047   0.935
E K  4L72A                428    0.332  68.292   0.630   0.005   0.262   0.733
B Y  4L72A                429    0.126  26.883   1.693   0.001   0.900   0.099
B Y  4L72A                430    0.018   3.847   1.528   0.001   0.959   0.040
B Q  4L72A                431    0.027   4.822   1.033   0.000   0.983   0.017
B L  4L72A                432    0.027   4.889   0.748   0.000   0.983   0.017
B R  4L72A                433    0.067  15.274   0.350   0.000   0.983   0.017
B C  4L72A                434    0.079  11.078  -0.369   0.001   0.959   0.040
B S  4L72A                435    0.231  27.050  -0.622   0.004   0.616   0.381
B G  4L72A                436    0.227  17.896  -1.482   0.004   0.138   0.858
B P  4L72A                437    0.279  39.548  -1.277   0.005   0.015   0.979
E G  4L72A                438    0.484  38.107  -1.192   0.005   0.015   0.979
E L  4L72A                439    0.396  72.489   0.111   0.005   0.015   0.979
B P  4L72A                440    0.144  20.405  -0.718   0.052   0.084   0.864
B L  4L72A                441    0.232  42.443  -0.643   0.021   0.451   0.528
B Y  4L72A                442    0.185  39.620  -0.065   0.018   0.846   0.136
B T  4L72A                443    0.082  11.304   0.807   0.011   0.918   0.071
B L  4L72A                444    0.050   9.118   0.994   0.006   0.962   0.032
B H  4L72A                445    0.129  23.429   0.427   0.001   0.959   0.040
B S  4L72A                446    0.117  13.689   0.211   0.001   0.900   0.099
B S  4L72A                447    0.225  26.323  -0.277   0.004   0.616   0.381
E V  4L72A                448    0.322  49.445  -0.109   0.004   0.085   0.910
E N  4L72A                449    0.751 109.873  -0.015   0.005   0.015   0.979
E D  4L72A                450    0.662  95.365  -1.301   0.005   0.015   0.979
E K  4L72A                451    0.585 120.293  -0.596   0.018   0.047   0.935
E G  4L72A                452    0.489  38.453   0.809   0.022   0.552   0.426
E L  4L72A                453    0.278  50.938   0.685   0.018   0.846   0.136
E R  4L72A                454    0.357  81.845   1.212   0.011   0.918   0.071
E V  4L72A                455    0.294  45.249   1.412   0.011   0.918   0.071
B L  4L72A                456    0.073  13.421   0.489   0.018   0.846   0.136
E E  4L72A                457    0.455  79.541  -0.062   0.023   0.655   0.322
E D  4L72A                458    0.450  64.773  -0.179   0.022   0.359   0.619
B N  4L72A                459    0.127  18.651  -0.370   0.052   0.084   0.864
E S  4L72A                460    0.600  70.320  -0.376   0.184   0.043   0.773
E A  4L72A                461    0.441  48.576  -0.419   0.184   0.043   0.773
B L  4L72A                462    0.172  31.438  -0.872   0.257   0.016   0.727
E D  4L72A                463    0.373  53.749  -0.301   0.339   0.016   0.645
E K  4L72A                464    0.580 119.327  -1.012   0.430   0.016   0.555
E M  4L72A                465    0.399  79.820  -0.286   0.430   0.016   0.555
B L  4L72A                466    0.153  28.014  -1.451   0.339   0.016   0.645
E Q  4L72A                467    0.627 111.911  -0.432   0.257   0.016   0.727
E N  4L72A                468    0.566  82.906  -1.433   0.113   0.043   0.844
B V  4L72A                469    0.238  36.627  -0.594   0.052   0.084   0.864
E Q  4L72A                470    0.565 100.855  -0.465   0.018   0.088   0.893
B M  4L72A                471    0.129  25.873   0.411   0.018   0.088   0.893
E P  4L72A                472    0.442  62.777   0.058   0.004   0.138   0.858
E S  4L72A                473    0.414  48.533   0.050   0.004   0.616   0.381
E K  4L72A                474    0.419  86.229   0.436   0.001   0.900   0.099
E K  4L72A                475    0.381  78.372   0.627   0.001   0.959   0.040
B L  4L72A                476    0.116  21.185   0.932   0.001   0.959   0.040
E D  4L72A                477    0.340  48.994   0.481   0.001   0.959   0.040
B F  4L72A                478    0.098  19.689   0.972   0.001   0.959   0.040
E I  4L72A                479    0.407  75.332   0.285   0.001   0.900   0.099
B I  4L72A                480    0.143  26.381  -0.364   0.004   0.514   0.481
E L  4L72A                481    0.546  99.973   0.085   0.004   0.197   0.799
E N  4L72A                482    0.689 100.943   0.066   0.005   0.015   0.979
E E  4L72A                483    0.587 102.619  -1.326   0.005   0.015   0.979
E T  4L72A                484    0.283  39.224   0.537   0.004   0.616   0.381
E K  4L72A                485    0.458  94.169   1.675   0.001   0.900   0.099
B F  4L72A                486    0.032   6.523   0.875   0.001   0.959   0.040
B W  4L72A                487    0.202  48.533   0.797   0.001   0.900   0.099
B Y  4L72A                488    0.023   4.958   0.215   0.001   0.959   0.040
B Q  4L72A                489    0.075  13.359   0.504   0.001   0.959   0.040
B M  4L72A                490    0.019   3.762   1.193   0.001   0.959   0.040
B I  4L72A                491    0.052   9.601   0.785   0.001   0.900   0.099
B L  4L72A                492    0.138  25.176   1.835   0.003   0.718   0.279
B P  4L72A                493    0.061   8.628   0.182   0.005   0.262   0.733
E P  4L72A                494    0.597  84.672   0.512   0.005   0.015   0.979
E H  4L72A                495    0.808 147.048  -0.537   0.005   0.015   0.979
B F  4L72A                496    0.175  35.203   0.013   0.005   0.015   0.979
E D  4L72A                497    0.556  80.062   1.169   0.018   0.019   0.964
E K  4L72A                498    0.663 136.276  -0.135   0.016   0.005   0.979
E S  4L72A                499    0.825  96.655  -0.263   0.016   0.005   0.979
E K  4L72A                500    0.450  92.565   0.498   0.005   0.015   0.979
E K  4L72A                501    0.525 107.972   1.584   0.005   0.045   0.951
B Y  4L72A                502    0.118  25.174   1.361   0.004   0.197   0.799
B P  4L72A                503    0.035   4.995   0.000   0.004   0.420   0.576
B L  4L72A                504    0.019   3.424   1.303   0.001   0.900   0.099
B L  4L72A                505    0.017   3.149   1.535   0.001   0.959   0.040
B L  4L72A                506    0.016   2.856   1.424   0.001   0.959   0.040
B D  4L72A                507    0.041   5.937   0.611   0.001   0.959   0.040
B V  4L72A                508    0.017   2.582   0.940   0.003   0.718   0.279
B Y  4L72A                509    0.047  10.044  -0.250   0.005   0.262   0.733
B A  4L72A                510    0.051   5.609  -2.455   0.018   0.047   0.935
B G  4L72A                511    0.051   3.974  -0.962   0.018   0.047   0.935
B P  4L72A                512    0.093  13.168  -1.566   0.018   0.047   0.935
B C  4L72A                513    0.148  20.751  -1.161   0.018   0.047   0.935
B S  4L72A                514    0.076   8.931  -1.909   0.018   0.088   0.893
B Q  4L72A                515    0.133  23.718  -2.101   0.021   0.279   0.699
B K  4L72A                516    0.216  44.390  -1.095   0.021   0.279   0.699
B A  4L72A                517    0.108  11.935  -0.499   0.021   0.279   0.699
E D  4L72A                518    0.280  40.391  -0.572   0.018   0.088   0.893
E T  4L72A                519    0.431  59.794  -1.870   0.018   0.047   0.935
B V  4L72A                520    0.270  41.560  -1.944   0.018   0.088   0.893
B F  4L72A                521    0.158  31.650  -2.397   0.019   0.141   0.840
B R  4L72A                522    0.151  34.671  -3.079   0.019   0.141   0.840
B L  4L72A                523    0.128  23.492  -2.758   0.018   0.088   0.893
B N  4L72A                524    0.206  30.158  -1.199   0.018   0.047   0.935
B W  4L72A                525    0.104  25.084  -1.301   0.717   0.014   0.269
B A  4L72A                526    0.129  14.260  -1.089   0.802   0.014   0.185
B T  4L72A                527    0.061   8.475  -0.138   0.879   0.010   0.111
B Y  4L72A                528    0.076  16.198   0.739   0.938   0.007   0.055
B L  4L72A                529    0.072  13.275   0.394   0.938   0.007   0.055
B A  4L72A                530    0.029   3.174   0.405   0.879   0.010   0.111
B S  4L72A                531    0.046   5.426  -0.157   0.802   0.014   0.185
B T  4L72A                532    0.196  27.241  -0.867   0.522   0.016   0.462
B E  4L72A                533    0.185  32.389   0.300   0.018   0.019   0.964
B N  4L72A                534    0.107  15.592  -1.158   0.005   0.045   0.951
B I  4L72A                535    0.053   9.824   0.552   0.004   0.514   0.481
B I  4L72A                536    0.019   3.570   1.211   0.001   0.959   0.040
B V  4L72A                537    0.021   3.182   1.597   0.000   0.983   0.017
B A  4L72A                538    0.015   1.675   1.551   0.000   0.983   0.017
B S  4L72A                539    0.047   5.485   0.815   0.001   0.959   0.040
B F  4L72A                540    0.019   3.853   1.128   0.002   0.816   0.182
B D  4L72A                541    0.055   7.926  -0.304   0.005   0.262   0.733
B G  4L72A                542    0.110   8.673  -1.575   0.004   0.085   0.910
B R  4L72A                543    0.122  27.915  -1.278   0.018   0.047   0.935
B G  4L72A                544    0.104   8.153  -3.318   0.005   0.045   0.951
B S  4L72A                545    0.100  11.732  -3.196   0.018   0.019   0.964
B G  4L72A                546    0.266  20.903  -1.378   0.058   0.017   0.925
E Y  4L72A                547    0.360  76.953  -2.607   0.109   0.005   0.886
B Q  4L72A                548    0.234  41.846  -0.138   0.176   0.004   0.820
B G  4L72A                549    0.036   2.857  -2.556   0.246   0.004   0.750
E D  4L72A                550    0.478  68.837   0.838   0.858   0.002   0.139
E K  4L72A                551    0.469  96.391   1.371   0.923   0.002   0.076
B I  4L72A                552    0.041   7.677   0.151   0.923   0.002   0.076
B M  4L72A                553    0.153  30.575   0.474   0.970   0.001   0.030
E H  4L72A                554    0.518  94.206   0.755   0.923   0.002   0.076
B A  4L72A                555    0.179  19.726   0.137   0.858   0.002   0.139
B I  4L72A                556    0.039   7.141  -2.027   0.782   0.003   0.216
E N  4L72A                557    0.312  45.691   0.318   0.694   0.003   0.303
E R  4L72A                558    0.572 130.896  -0.356   0.502   0.002   0.495
E R  4L72A                559    0.548 125.538   0.157   0.406   0.004   0.590
B L  4L72A                560    0.248  45.336  -0.182   0.246   0.004   0.750
E G  4L72A                561    0.390  30.669  -0.716   0.053   0.005   0.942
E T  4L72A                562    0.608  84.371  -0.329   0.018   0.019   0.964
E F  4L72A                563    0.294  58.946   0.598   0.053   0.043   0.903
E E  4L72A                564    0.276  48.182   0.367   0.181   0.016   0.803
B V  4L72A                565    0.114  17.568   0.914   0.923   0.002   0.076
E E  4L72A                566    0.496  86.634   1.585   0.970   0.001   0.030
B D  4L72A                567    0.037   5.346   0.048   0.970   0.001   0.030
B Q  4L72A                568    0.023   4.019   1.479   0.988   0.000   0.012
B I  4L72A                569    0.089  16.391   0.692   0.970   0.001   0.030
E E  4L72A                570    0.274  47.903   1.791   0.970   0.001   0.030
B A  4L72A                571    0.016   1.796   1.305   0.970   0.001   0.030
B A  4L72A                572    0.029   3.174   0.998   0.970   0.001   0.030
E R  4L72A                573    0.410  93.776   2.121   0.988   0.000   0.012
B Q  4L72A                574    0.171  30.558   1.868   0.988   0.000   0.012
B F  4L72A                575    0.019   3.713   1.241   0.970   0.001   0.030
B S  4L72A                576    0.219  25.620   1.041   0.970   0.001   0.030
E K  4L72A                577    0.849 174.598   1.566   0.858   0.002   0.139
E M  4L72A                578    0.296  59.190   2.124   0.246   0.004   0.750
E G  4L72A                579    0.564  44.347  -0.539   0.016   0.005   0.979
B F  4L72A                580    0.245  49.151  -0.494   0.005   0.015   0.979
B V  4L72A                581    0.046   7.086  -0.736   0.018   0.088   0.893
B D  4L72A                582    0.183  26.341   0.941   0.018   0.047   0.935
E N  4L72A                583    0.496  72.556   0.385   0.058   0.017   0.925
E K  4L72A                584    0.588 120.910   0.840   0.058   0.017   0.925
B R  4L72A                585    0.120  27.388   1.245   0.056   0.142   0.802
B I  4L72A                586    0.020   3.756   1.142   0.087   0.683   0.230
B A  4L72A                587    0.032   3.504   0.377   0.018   0.846   0.136
B I  4L72A                588    0.020   3.718   0.501   0.018   0.846   0.136
B W  4L72A                589    0.067  16.162   0.866   0.018   0.846   0.136
B G  4L72A                590    0.031   2.479  -1.631   0.004   0.514   0.481
B W  4L72A                591    0.104  25.084  -0.040   0.004   0.197   0.799
B S  4L72A                592    0.052   6.083  -1.687   0.053   0.043   0.903
B Y  4L72A                593    0.110  23.550  -0.662   0.782   0.003   0.216
B G  4L72A                594    0.041   3.227  -1.031   0.923   0.002   0.076
B G  4L72A                595    0.033   2.621  -0.655   0.970   0.001   0.030
B Y  4L72A                596    0.059  12.523  -0.268   0.970   0.001   0.030
B V  4L72A                597    0.033   5.149   0.103   0.970   0.001   0.030
B T  4L72A                598    0.026   3.551   0.694   0.970   0.001   0.030
B S  4L72A                599    0.029   3.387  -0.575   0.970   0.001   0.030
B M  4L72A                600    0.086  17.189   0.227   0.970   0.001   0.030
B V  4L72A                601    0.030   4.626  -0.580   0.970   0.001   0.030
B L  4L72A                602    0.030   5.548   0.138   0.923   0.002   0.076
B G  4L72A                603    0.058   4.572  -1.035   0.782   0.003   0.216
E S  4L72A                604    0.314  36.754   0.333   0.406   0.004   0.590
E G  4L72A                605    0.330  25.932   0.211   0.053   0.005   0.942
B S  4L72A                606    0.247  28.948  -0.402   0.005   0.015   0.979
E G  4L72A                607    0.451  35.525  -0.698   0.018   0.047   0.935
B V  4L72A                608    0.110  16.907   0.874   0.021   0.279   0.699
B F  4L72A                609    0.032   6.342   0.330   0.003   0.718   0.279
B K  4L72A                610    0.105  21.681   0.530   0.011   0.918   0.071
B C  4L72A                611    0.018   2.569   1.065   0.006   0.962   0.032
B G  4L72A                612    0.018   1.385   0.974   0.011   0.918   0.071
B I  4L72A                613    0.026   4.755   0.790   0.011   0.918   0.071
B A  4L72A                614    0.023   2.524   0.181   0.021   0.756   0.223
B V  4L72A                615    0.084  12.911  -1.631   0.021   0.451   0.528
B A  4L72A                616    0.051   5.576   0.132   0.020   0.205   0.775
B P  4L72A                617    0.043   6.088  -0.156   0.019   0.141   0.840
B V  4L72A                618    0.032   4.934  -1.071   0.056   0.142   0.802
B S  4L72A                619    0.063   7.337  -0.702   0.113   0.087   0.800
B R  4L72A                620    0.056  12.778  -1.271   0.113   0.043   0.844
B W  4L72A                621    0.080  19.336   0.120   0.257   0.016   0.727
B E  4L72A                622    0.222  38.836  -0.389   0.268   0.043   0.689
B Y  4L72A                623    0.224  47.805  -0.933   0.268   0.043   0.689
B Y  4L72A                624    0.098  20.985  -0.545   0.184   0.043   0.773
E D  4L72A                625    0.345  49.729  -1.519   0.181   0.016   0.803
E S  4L72A                626    0.277  32.418  -1.808   0.184   0.043   0.773
B V  4L72A                627    0.197  30.233  -1.034   0.184   0.043   0.773
B Y  4L72A                628    0.116  24.875   0.110   0.268   0.043   0.689
B T  4L72A                629    0.181  25.105  -0.697   0.354   0.048   0.598
E E  4L72A                630    0.347  60.621  -1.091   0.561   0.047   0.393
E R  4L72A                631    0.333  76.257  -0.492   0.522   0.016   0.462
B Y  4L72A                632    0.115  24.490  -1.301   0.522   0.016   0.462
B M  4L72A                633    0.243  48.524  -1.286   0.339   0.016   0.645
E G  4L72A                634    0.550  43.277  -2.270   0.181   0.016   0.803
B L  4L72A                635    0.281  51.451  -0.963   0.058   0.017   0.925
E P  4L72A                636    0.410  58.151  -1.700   0.018   0.019   0.964
E T  4L72A                637    0.439  60.875  -0.302   0.005   0.015   0.979
E P  4L72A                638    0.246  34.922   0.132   0.406   0.004   0.590
E E  4L72A                639    0.493  86.197  -0.461   0.782   0.003   0.216
E D  4L72A                640    0.558  80.422   0.358   0.600   0.003   0.397
B N  4L72A                641    0.123  18.051  -0.476   0.522   0.016   0.462
B L  4L72A                642    0.162  29.717   0.541   0.923   0.002   0.076
E D  4L72A                643    0.540  77.814   1.362   0.970   0.001   0.030
B H  4L72A                644    0.209  38.108   1.662   0.970   0.001   0.030
B Y  4L72A                645    0.023   4.915   0.967   0.970   0.001   0.030
E R  4L72A                646    0.292  66.868   0.931   0.938   0.007   0.055
E N  4L72A                647    0.641  93.798   0.489   0.802   0.014   0.185
B S  4L72A                648    0.206  24.178   0.327   0.339   0.016   0.645
B T  4L72A                649    0.080  11.096  -0.605   0.058   0.017   0.925
B V  4L72A                650    0.105  16.077  -0.682   0.522   0.016   0.462
B M  4L72A                651    0.130  26.033  -0.002   0.622   0.015   0.363
E S  4L72A                652    0.397  46.482   0.445   0.622   0.015   0.363
B R  4L72A                653    0.244  55.853   0.439   0.622   0.015   0.363
B A  4L72A                654    0.037   4.044  -1.375   0.622   0.015   0.363
E E  4L72A                655    0.611 106.812   0.303   0.430   0.016   0.555
E N  4L72A                656    0.550  80.476   0.935   0.246   0.004   0.750
B F  4L72A                657    0.121  24.305  -0.039   0.181   0.016   0.803
E K  4L72A                658    0.527 108.404   0.282   0.115   0.016   0.868
E Q  4L72A                659    0.489  87.335  -0.055   0.018   0.019   0.964
B V  4L72A                660    0.102  15.739   1.361   0.018   0.019   0.964
B E  4L72A                661    0.125  21.820   0.354   0.019   0.141   0.840
B Y  4L72A                662    0.031   6.625   0.743   0.002   0.816   0.182
B L  4L72A                663    0.018   3.351   1.593   0.001   0.959   0.040
B L  4L72A                664    0.015   2.692   1.637   0.001   0.959   0.040
B I  4L72A                665    0.021   3.811   1.198   0.001   0.900   0.099
B H  4L72A                666    0.024   4.329   1.080   0.002   0.816   0.182
B G  4L72A                667    0.032   2.550  -0.889   0.005   0.336   0.660
B T  4L72A                668    0.205  28.378  -0.248   0.018   0.047   0.935
E A  4L72A                669    0.439  48.345  -0.281   0.018   0.047   0.935
B D  4L72A                670    0.039   5.663  -1.011   0.018   0.019   0.964
E D  4L72A                671    0.413  59.513  -0.315   0.018   0.047   0.935
E N  4L72A                672    0.269  39.367  -0.382   0.004   0.085   0.910
B V  4L72A                673    0.024   3.704   1.119   0.004   0.085   0.910
B H  4L72A                674    0.165  29.941   1.186   0.005   0.045   0.951
B F  4L72A                675    0.110  22.097  -0.227   0.717   0.014   0.269
B Q  4L72A                676    0.228  40.757   0.215   0.858   0.002   0.139
B Q  4L72A                677    0.094  16.753  -0.109   0.879   0.010   0.111
B S  4L72A                678    0.019   2.203   0.230   0.923   0.002   0.076
B A  4L72A                679    0.168  18.503   0.549   0.970   0.001   0.030
E Q  4L72A                680    0.337  60.224   1.960   0.970   0.001   0.030
B I  4L72A                681    0.025   4.680   1.107   0.970   0.001   0.030
B S  4L72A                682    0.045   5.251   0.709   0.970   0.001   0.030
E K  4L72A                683    0.369  75.862   2.063   0.970   0.001   0.030
B A  4L72A                684    0.153  16.828   1.569   0.970   0.001   0.030
B L  4L72A                685    0.022   4.010   1.015   0.970   0.001   0.030
E V  4L72A                686    0.382  58.744   1.886   0.923   0.002   0.076
E D  4L72A                687    0.715 102.974   1.345   0.858   0.002   0.139
E V  4L72A                688    0.355  54.579   1.231   0.246   0.004   0.750
E G  4L72A                689    0.863  67.879   0.297   0.003   0.003   0.994
B V  4L72A                690    0.179  27.435   1.297   0.005   0.015   0.979
E D  4L72A                691    0.431  62.064   0.783   0.004   0.138   0.858
B F  4L72A                692    0.067  13.427   0.754   0.002   0.816   0.182
B Q  4L72A                693    0.176  31.362   1.030   0.001   0.959   0.040
B A  4L72A                694    0.042   4.661   0.775   0.001   0.959   0.040
B M  4L72A                695    0.045   9.105   1.304   0.001   0.959   0.040
B W  4L72A                696    0.085  20.491   0.656   0.001   0.959   0.040
B Y  4L72A                697    0.058  12.395   1.006   0.003   0.718   0.279
E T  4L72A                698    0.271  37.560   0.716   0.004   0.138   0.858
E D  4L72A                699    0.523  75.422  -1.172   0.018   0.019   0.964
E E  4L72A                700    0.339  59.153  -0.767   0.018   0.019   0.964
E D  4L72A                701    0.374  53.937  -1.361   0.018   0.019   0.964
B H  4L72A                702    0.071  12.988  -1.581   0.053   0.043   0.903
E G  4L72A                703    0.275  21.650  -1.643   0.018   0.088   0.893
B I  4L72A                704    0.192  35.576  -1.109   0.018   0.088   0.893
E A  4L72A                705    0.385  42.416  -0.929   0.018   0.047   0.935
E S  4L72A                706    0.383  44.864  -0.321   0.018   0.019   0.964
E S  4L72A                707    0.425  49.857  -0.606   0.339   0.016   0.645
E T  4L72A                708    0.590  81.875  -0.700   0.622   0.015   0.363
B A  4L72A                709    0.209  23.021  -0.471   0.802   0.014   0.185
B H  4L72A                710    0.160  29.177   0.821   0.975   0.003   0.022
B Q  4L72A                711    0.177  31.666   0.991   0.975   0.003   0.022
E H  4L72A                712    0.310  56.316   1.750   0.970   0.001   0.030
B I  4L72A                713    0.079  14.652   0.934   0.970   0.001   0.030
B Y  4L72A                714    0.063  13.399   0.924   0.970   0.001   0.030
E T  4L72A                715    0.316  43.760   1.661   0.970   0.001   0.030
B H  4L72A                716    0.233  42.346   1.534   0.970   0.001   0.030
B M  4L72A                717    0.035   6.923   0.576   0.970   0.001   0.030
B S  4L72A                718    0.079   9.282   0.755   0.970   0.001   0.030
E H  4L72A                719    0.352  64.029   1.706   0.970   0.001   0.030
B F  4L72A                720    0.090  18.143   1.034   0.970   0.001   0.030
B I  4L72A                721    0.029   5.439   0.712   0.988   0.000   0.012
E K  4L72A                722    0.275  56.547   1.218   0.970   0.001   0.030
E Q  4L72A                723    0.595 106.338   1.550   0.923   0.002   0.076
B C  4L72A                724    0.234  32.882   0.697   0.858   0.002   0.139
B F  4L72A                725    0.081  16.337  -0.488   0.502   0.002   0.495
E S  4L72A                726    0.692  81.056   0.468   0.053   0.005   0.942
E L  4L72A                727    0.331  60.679   0.699   0.016   0.005   0.979
E P  4L72A                728    0.701  99.415  -1.527   0.000   0.001   0.999

Explain the output.  Go back.
```

---
